# Comprehensive Analysis of Mandibular Residual Asymmetry after Bilateral Sagittal Split Ramus Osteotomy Correction of Menton Point Deviation

**DOI:** 10.1371/journal.pone.0161601

**Published:** 2016-08-29

**Authors:** Han Lin, Ping Zhu, Qiuping Lin, Xiaoqiong Huang, Yue Xu, Xiaoping Yang

**Affiliations:** 1 Department of Oral and Maxillofacial Surgery, Guanghua School of Stomatology, Hospital of Stomatology, Sun Yat-sen University, Guangdong Provincial Key Laboratory of Stomatology, Guangzhou, China; 2 Department of Orthodontics, Guanghua School of Stomatology, Hospital of Stomatology, Sun Yat-sen University, Guangdong Provincial Key Laboratory of Stomatology, Guangzhou, China; Medical University of South Carolina, UNITED STATES

## Abstract

**Purpose:**

Facial asymmetry often persists even after mandibular deviation corrected by the bilateral sagittal split ramus osteotomy (BSSRO) operation, since the reference facial sagittal plane for the asymmetry analysis is usually set up before the mandibular menton (Me) point correction. Our aim is to develop a predictive and quantitative method to assess the true asymmetry of the mandible after a midline correction performed by a virtual BSSRO, and to verify its availability by evaluation of the post-surgical improvement.

**Patients and Methods:**

A retrospective cohort study was conducted at the Hospital of Stomatology, Sun Yat-sen University (China) of patients with pure hemi-mandibular elongation (HE) from September 2010 through May 2014. Mandibular models were reconstructed from CBCT images of patients with pre-surgical orthodontic treatment. After mandibular de-rotation and midline alignment with virtual BSSRO, the elongation hemi-mandible was virtually mirrored along the facial sagittal plane. The residual asymmetry, defined as the superimposition and boolean operation of the mirrored elongation side on the normal side, was calculated, including the volumetric differences and the length of transversal and vertical asymmetry discrepancy. For more specific evaluation, both sides of the hemi-mandible were divided into the symphysis and parasymphysis (SP), mandibular body (MB), and mandibular angle (MA) regions. Other clinical variables include deviation of Me point, dental midline and molar relationship. The measurement of volumetric discrepancy between the two sides of post-surgical hemi-mandible were also calculated to verify the availability of virtual surgery. Paired t-tests were computed and the *P* value was set at .05.

**Results:**

This study included 45 patients. The volume differences were 407.8±64.8 mm^3^, 2139.1±72.5 mm^3^, and 422.5±36.9 mm^3^; residual average transversal discrepancy, 1.9 mm, 1.0 mm, and 2.2 mm; average vertical discrepancy, 1.1 mm, 2.2 mm, and 2.2 mm (before virtual surgery). The post-surgical volumetric measurement showed no statistical differences between bilateral mandibular regions.

**Conclusions:**

Mandibular asymmetry persists after Me point correction. A 3D quantification of mandibular residual asymmetry after Me point correction and mandible de-rotation with virtual BSSRO sets up a true reference mirror plane for comprehensive asymmetry assessment of bilateral mandibular structure, thereby providing an accurate guidance for orthognathic surgical planning.

## Introduction

Facial symmetry is an essential prerequisite of a successful reconstructive and aesthetic plastic surgery. Pure hemi-mandibular elongation is often manifested as deviation of the mandibular menton (Me) point and is usually accompanied by functional abnormalities, inducing a considerable impact on the patient’s psychological wellbeing [1]. In lieu of the geometric complexity of dentition and the bony structures and soft tissue of the face, the correction of severe facial asymmetry is an extremely challenging prospect in orthognathic surgery [2].

Among the current surgical approaches, bilateral sagittal split ramus osteotomy (BSSRO) is one of the most common orthognathic procedures: it involves the rotation and movement of the osteotomized segments of the mandible with sufficient flexibility and safety of the mandibular nerve [3–5]. Orthognathic surgery is performed with correction of the mandibular rotation first, followed by marginal and surface modification [6]. Some previous reports evaluate the mandibular asymmetry by mirroring the semi-mandible with the facial mid-sagittal plane without considering secondary mandibular rotation after Me point correction, while others assess the mandibular asymmetry before the midline correction [7–9]. The comprehensive pre-surgical analysis is of significance, as it can aid in planning the deviation correction and residual asymmetry modification at the same time. As a result, it is unreasonable to use the facial sagittal plane as the reference plane for the symmetry analysis before surgically correcting the midline and de-rotating the mandible [10].

Over the past decades, the introduction of three-dimensional (3D) measurement technology has significantly improved the surgical planning of the mandibular deviation correction as well as the prediction of the treatment outcome. However, today, few statistical reports on the volume or length differences and specific locations of asymmetry on patients with mandibular deviation have been published. In most cases, the secondary mandibular morphology surgical correction after BSSRO is made on the basis of the surgeons’ clinical experience, rather than a precisely quantitative assessment of the characteristics of mandibular deviation [11]. This makes it difficult to predict ideal symmetry after the surgery. In other words, a predictively quantitative analysis of bilateral mandibular symmetry in patients with mandibular deviation after virtual BSSRO yields a much better clinical reference value than an evaluation before bony segment de-rotation in deviation modification surgery.

The purpose of this study is to quantitatively predict the mandibular asymmetry in patients with mandibular deviation after Me point virtual correction. We hypothesize that the mirroring reference plane differed from the mid-sagittal plane, which causes the residual asymmetry of bilateral mandibular structure after Me point correction. The specific aims of the study are to measure the extent (volume and length) of discrepancy and specific location of mandibular asymmetry as well as verification by post-surgical outcomes.

## Materials and Methods

### Study design/sample

To address the research purpose, this study is designed as a retrospective investigation. The study population is composed of patients diagnosed with pure hemi-mandibular elongation (HE) at the Hospital of Stomatology, Sun Yat-sen University, Guangzhou, China from September 2010 through May 2014. The study protocol was approved by the institutional review board at the Sun Yat-sen University and all participants signed an informed consent agreement. To be included in the study sample, patients had to meet the following inclusion criteria: (a) ANB angle of <0°, (b) dental midline deviation of ≥ 2 mm, and (c) Me point deviation of ≥ 4 mm. Patients who had a history of proven fracture, surgery, or trauma of the mandible were excluded as study subjects.

### Clinical examination

Data on the following parameters were collected for each patient:

1. Deviation of Me point: Deviation of the Me point was measured as the distance between the Me point and the facial midline. The facial midline was defined as the perpendicular bisector of the line drawn between the centers of the right and left pupils.
2. Deviation of the dental midline: Deviation of the dental midlines was measured as the horizontal distance between the mesial contact points of the maxillary central incisors and mandibular central incisors.
3. Inclination of the maxillary occlusal plane: To determine the inclination of the maxillary occlusal plane, patients were required to bite onto a tongue blade; then, the slant in the occlusal plane was detected as the angle between the blade and the inter-pupillary plane.
4. Molar relationship.
5. ANB angle.

### Virtual surgery procedure

**Fig 1:**
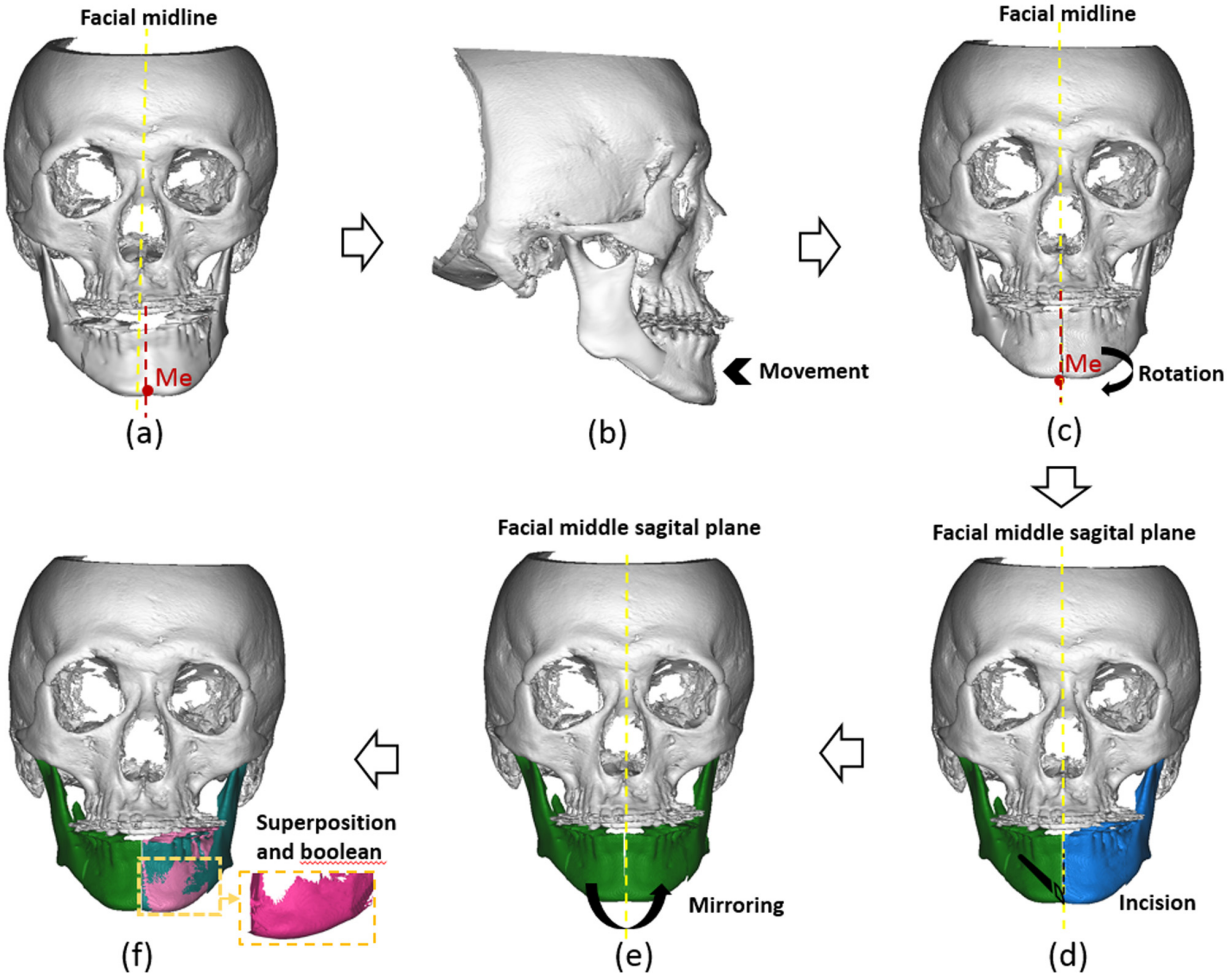
Procedure for measurement of the mandibular residual asymmetry: (a) Incision design and measurement before surgery; (b) Mandible segment backwards to normal maxillo-mandibular relationship; (c) Me point—facial midline alignment by mandibular de-rotation; (d) Segmentation along the facial middle sagittal plane, green color represents the elongation hemi-mandible; blue color represents the normal hemi-mandible; (e) Mirroring of elongation hemi-mandible along facial middle sagittal plane; (f) Superimposition and boolean operation of mirrored elongation hemi-mandible and normal side, pink color in the box represents the residual asymmetry between the two sides of the mandible.

Before surgery, all of the patients were received pre-surgical orthodontic treatment. Alignment and coordination of the maxilla-mandibular dental arch width were performed to ascertain that the Me point could be adjusted to a precise position. CBCT images of patients with pre-surgical orthodontic treatment were acquired using the DCT Pro CBCT device (Vatech Co., Ltd., Hwasung, Korea), while 3D reconstruction was performed using the Mimics^™^ program (Materialise’s interactive medical image control system, Mimics, 14.0; Materialise, Leuven, Belgium). Virtual BSSRO was then performed on the 3D craniofacial models. The distal segments were separated from the proximal segment on both sides of the mandible, and the proximal segment was rotated and shifted backwards to achieve normal jaw relationship and midline alignment. Maxillary surgery (Lefort I) was additionally performed if an inclination of the occlusal plane was noted. After correction of the Me point, a real virtual mirroring plane (a sagittal plane passing through the facial midline and corrected Me point) for residual asymmetry measurement was created. The elongation side of the hemi-mandible was mirrored to create a mirrored model. Residual asymmetry was defined as the superimposition and boolean calculation of mirrored elongation side on the normal side (Fig 1).

### Residual asymmetry measurement

**Fig 2:**
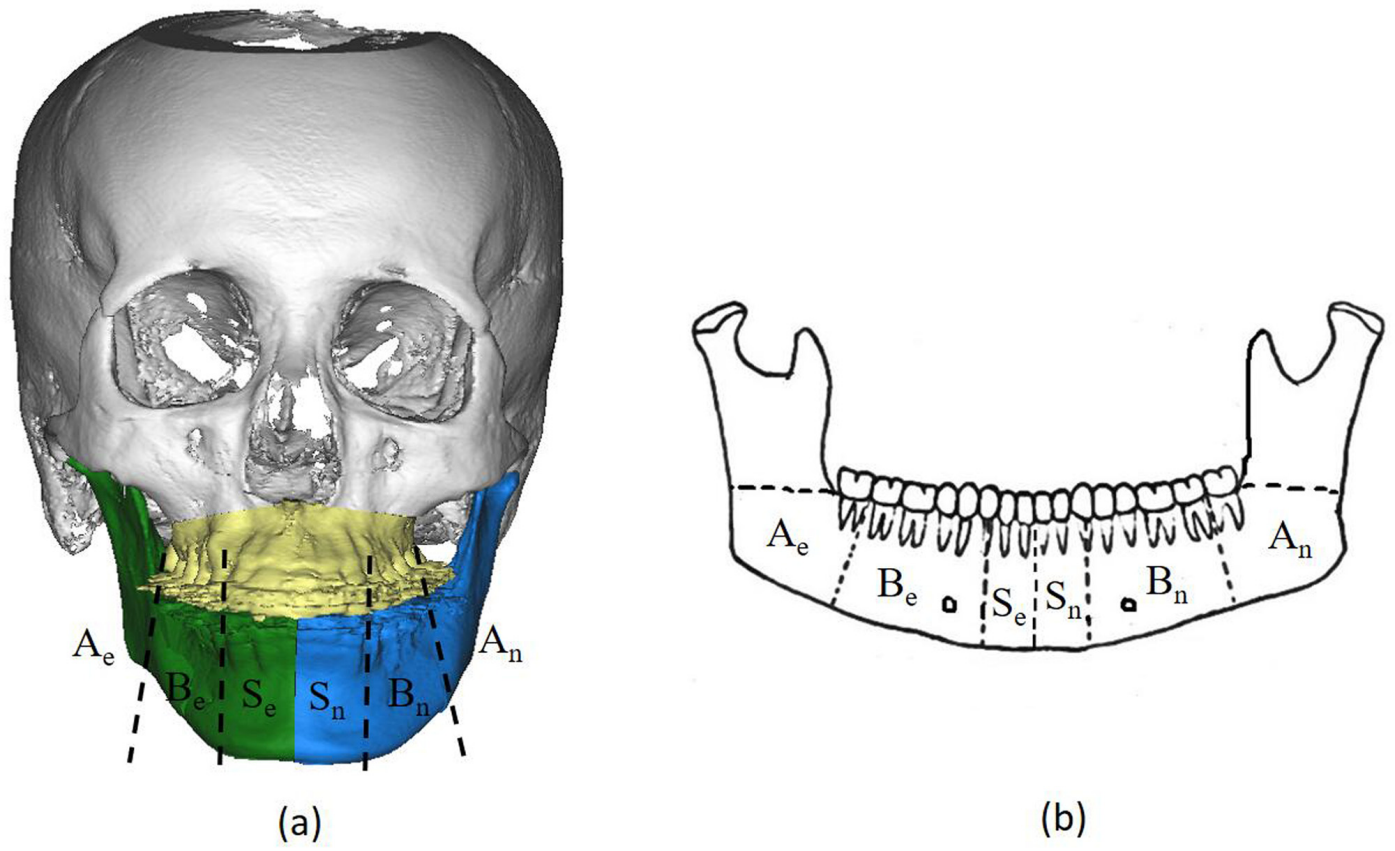
Mandibular division: (a) Both sides of hemi-mandible were divided into three regions respectively after virtual Me point correction; (b) Schematic diagram of three mandibular regions: S, Symphysis and parasymphysis mental region; M, Mandibular body region; A, Mandibular angle region; the subscripts e, n represent elongation and normal side.

**Fig 3:**
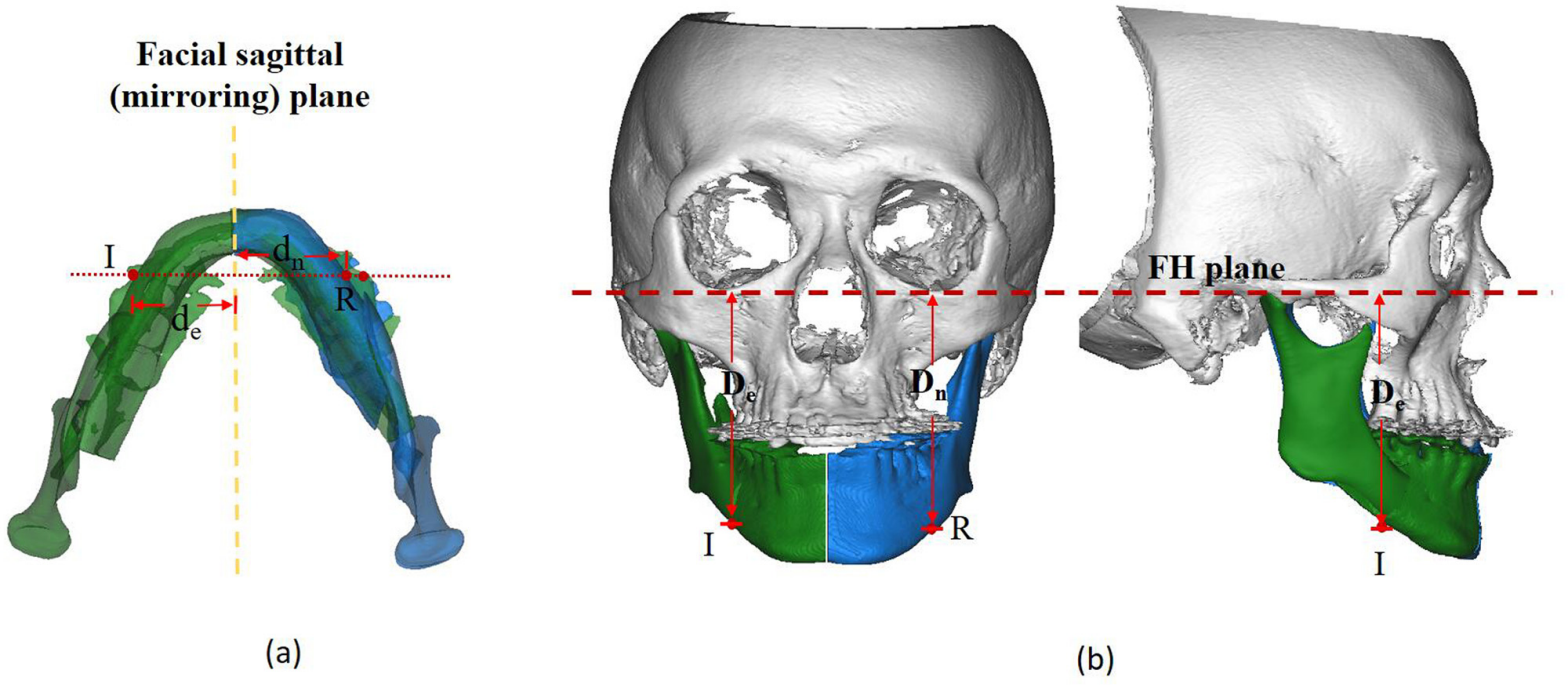
3D measurement of the mandibular asymmetry (e.g., MB region): (a) Evaluation of the extent of transversal asymmetry (cross-sectional view). Point I represents the point of inflection in the asymmetry site on elongation side, while point R represents the contralateral reference point on the normal side. The distance from points I/R to the facial middle sagittal plane were measured for transversal asymmetry extent evaluation, indicated as d_e_ and d_n_ respectively; (b) Evaluation of the extent of vertical asymmetry (front and lateral view). The distance from point I/R to the Frankfort horizontal (FH) plane were measured to determine the extent of vertical asymmetry, indicated as D_e_/D_n_.

To ensure a thorough assessment of mandibular asymmetry, both sides of therepositioned mandible is divided into three parts: the SP region comprising of the symphysis and parasymphysis, MB region comprising of the mandibular body, and the MA region comprising of the mandibular angle (the SP, MB, and MA regions are abbreviated as S, B, and A, respectively; the elongation and normal sides are represented by the subscripts *e* and *n*, respectively) [12] (Fig 2). The volume (mm^3^) of the residual asymmetry was measured using the Mimics^™^ automatic function. For measurement of transversal length discrepancy, on the elongation side, the extent of transversal length (d_e_) is defined as the distance from the inflecting point (point I) at the site of asymmetry to the facial sagittal plane. For comparison, on the normal side, the distance (d_n_) is measured from reference point (R point) to the facial sagittal plane. The vertical asymmetry discrepancy is defined as the length difference between distance D_e_ (measured from point I to Frankfort horizontal (FH) plane) and distance D_n_ (measured from point R to FH plane) (Fig 3).

### Post-surgical verification

The availability of the virtual surgery is verified by post-surgical measurement. Prior to surgery, a computer-guided digital template for each patient was designed and fabricated to help perform the precise osteotomy and to retain the modified bony segments in the proper position of mandible in clinic as in the virtual surgical planning. CBCT images of patients were acquired six months after surgery. The reconstructed mandible model is divided into two hemi-mandibles along the facial sagittal plane, followed by volumetric measurement (S2 Fig). Clinical examination parameters are the same as mentioned above.

### Data Analysis

The values of volume and length discrepancy of mandibular asymmetry are presented as mean ± standard deviation. The measurements were processed and analyzed using SPSS Inc. Released 2007, SPSS for Windows, Version 16.0. (SPSS Inc., Chicago, IL). The paired t-test was used to calculate the statistical significance of the difference between the volume and length of asymmetry in the SP, MB, and MA regions of the mirrored elongation and normal sides. A confidence level of a *P* value less than .05 was accepted as significant.

## Results

A total of 45 pure HE patients (23 males, age, 23±4 yrs and 22 females, age, 25±5 yrs) were reviewed retrospectively.

The average volumetric discrepancy after virtual Me point correction in the SP, MB, and MA regions were 407.8±64.8 mm^3^, 2139.1±72.5 mm^3^, and 422.5±36.9mm^3^, respectively. All with significant differences (*P* < 0.05), while no statistical difference was found between all of the bilateral mandibular regions in the post-surgical volumetric measurement (Table 1). Typical cases of mandibular residual asymmetry in different regions after virtual Me point correction are shown in S1 Fig.

**Table 1:** The volumetric measurement of the mandibular regions after virtual Me point correction and after clinical orthognathic surgery.

	Virtual Me point correction group			Post-surgical group		
	SP (mm^3^)	MB (mm^3^)	MA (mm^3^)	SP (mm^3^)	MB (mm^3^)	MA (mm^3^)
Elongation side	3899.5±244.8	12189.2±303.1	2310.8±175.4	3479.3±189.7	10184.4±268.0	1951.2±204.1
Normal side	3491.7±190.9	10050.2±241.0	1888.3±163.3	3418.3±202.5	10076.3±313.3	1883.0±139.0
Volume difference	407.8±64.8	2139.1±72.5	422.5±36.9	61.0±247.5	108.2±405.2	68.2±231.5
*P* value	<0.05	<0.05	<0.05	0.106	0.080	0.054

n = 45; Me, menton; SP, symphysis and parasymphysis; MB, mandibular body; MA, mandibular angle;

The average transversal and vertical discrepancy of the asymmetry sites in the SP, MB, and MA regions were 1.9 mm transversally and 1.1 mm vertically, 1.0 mm transversally and 2.2 mm vertically, 2.2 mm transversally and 2.2 mm vertically, respectively, and all with significant differences (*P* < 0.05), as shown in Tables 2 and 3.

**Table 2:** Measurement of the transversal discrepancy of asymmetry site in SP, MB, and MA regions after virtual Me point correction.

	SP (mm)	MB (mm)	MA (mm)
Transversal length on elongation side (d_e_)	6.6±2.1	7.2±1.8	9.3±2.8
Transversal length on normal side (d_n_)	4.7±1.2	6.2±1.8	7.0±1.9
Transversal discrepancy	1.9±1.2	1.0±0.6	2.2±1.1
*P* value	<0.05	<0.05	<0.05

n = 45; Me, menton; SP, symphysis and parasymphysis; MB, mandibular body; MA, mandibular angle;

**Table 3:** Measurement of the vertical discrepancy of asymmetry site in SP, MB, and MA regions after virtual Me point correction.

	SP (mm)	MB (mm)	MA (mm)
Vertical length on elongation side (D_e_)	79.4±3.8	77.6±2.7	72.8±2.4
Vertical length on normal side (D_n_)	78.2±3.5	75.4±2.7	70.8±2.8
Vertical discrepancy	1.1±1.5	2.2±2.6	2.0±1.7
*P* value	<0.05	<0.05	<0.05

n = 45; Me, menton; SP, symphysis and parasymphysis; MB, mandibular body; MA, mandibular angle;

The results of the clinical examinations before virtual surgery and after surgery are shown in the S1 Table. The deviation of the Me point and dental midline before virtual surgery were 5.4±0.9 mm and 2.9±0.6 mm, respectively. While, the inclination of the maxillary occlusal plane was 6.1±1.3°. Among the 55 patients, 16 were positive for inclination of the occlusal plane, indicating an incidence of 29.1%. The average ANB angle was measured to be -5.1±1.7°. The post-surgical measurement results show that all of the parameters were corrected to normal range.

## Discussion

This is the first article reporting the statistical analysis of extent (volume and length discrepancy) of residual asymmetry on patients with mandibular deviation. Due to the complexity of the mandibular U-shaped morphology, menton deviation is usually accompanied by asymmetry of other parts of the mandible [6]. A correct reference plane is the basis of a comprehensive asymmetry assessment. In our study, an actual clinical sagittal reference plane passing through the facial midline and Me point was created for quantitative assessment of bilateral mandibular asymmetry after the Me point correction with the virtual BSSRO procedure.

To ensure a thorough assessment of mandibular asymmetry, the mandible is divided into three anatomical regions according to the characteristics of each region and the site of the surgical incision (Fig 2) [12]. In our previous research, volumetric measurement has been proved as a sensitive method for mandibular asymmetry assessment [13]. As shown in Table 1, the volume of the elongation side in all three mandibular regions was significantly greater than those in the contralateral side, while the volumetric discrepancy in the MB region was greatest. As the major part of the mandible contains the mandibular nerves, the MB region can pose many challenges in achieving symmetry of shape and curvature on the lower edge of the mandible. Our results showed agreement with a previous report demonstrating statistical differences of the surface distance between the ramus, mandibular body and the symphysis area on bilateral sides [14].

The major strength of this study is the revelation of the statistical analysis of residual asymmetry of the mandible, including the location and extent, based on a corrected mirrored reference plane. The differences of transversal and vertical measurement between different regions could guide the specific bony regions modification during the clinical orthognathic surgery. Putting together the results obtained for the deviation in the vertical and transversal directions in the different regions of the mandible, it could be concluded that for the SP region, the discrepancy in the transversal direction on the deviated side was a major cause of asymmetry (1.9 mm transversely and 1.1 mm vertically). This indicates that in clinical practice, for residual asymmetry in the SP region, outer bone cortex grinding [15] (for considerable transversal discrepancy) or splitting corticectomy (for minimal transversal discrepancy) would be a preferable procedure to inferior mandible border corticectomy. On the contrary, for the MB region, asymmetry is mainly caused by vertical discrepancy rather than transversal discrepancy (1.0 mm transversely and 2.2 mm vertically). Therefore, the suitable approach in deviations of the MB region would be full-thickness inferior mandible border corticectomy followed by outer cortex grinding or splitting corticectomy [11]. In the case of the MA region, both vertical and transverse discrepancy contributed to the asymmetry (2.2 mm transversely and 2.2 mm vertically). Hence, the combination of the above surgical methods would be necessary to achieve an ideal outcome in the modification of the MA region.

From the result of our post-surgical measurement, it can be seen that a high degree of consistency may be found between virtual BSSRO planning and clinical post-operation outcome, confirming that the residual asymmetry analysis services as an accurate guidance for orthognathic surgery. This study contains weaknesses: one is the complexity of the length measurement index which may hinder its application to manual landmark location. The application of automatic computer measurement of distance discrepancy could make up for this drawback.

## Conclusion

This study supports our finding that the mandibular asymmetry persists after the Me point correction. Me point correction with virtual BSSRO sets up a true reference mirrored plane for asymmetry assessment of bilateral mandibular structure, providing comprehensive planning of orthognathic surgery in patients with mandibular deviation. The variation in the location and extent of mandibular asymmetry would help orthognathic surgeons in making appropriate choices for modification operations along with the regular BSSRO, enabling correction of asymmetry in a single surgery. This would decrease the possibility of follow-up mandibular modification operations.

## Supporting Information

S1 Fig Typical cases of the mandibular residual asymmetry in different regions. the three models in the first row represent the stage before virtual BSSRO surgery (BSSRO with/without Lefort I operation). The second row of the models represent after virtual Me point correction, and mirroring and superimposition of the hemi-mandible. Pink color represents the residual asymmetry between both sides of the mandible. (a) SP region asymmetry: asymmetry mainly exist in the SP region; (b) MB region asymmetry: asymmetry mainly exist in the MB region; (c) MA region asymmetry: asymmetry mainly exist in the MA region.
(TIF)

S2 Fig Example of a typical case for evaluation method verification. (a) Three dimensional model with pre-surgical orthodontic treatment (before virtual BSSRO surgery); (b) Three dimensional model after virtual BSSRO surgery; (c) Three dimensional post-surgical model. The volumetric differences between both sides of hemi-mandible were calculated to verify the outcome consistency between surgery and virtual surgery.
(TIF)

S1 Table The mean values of clinical examination after virtual Me point correction and after clinical orthognathic surgery. (DOCX)

S1 Raw measurement data (ZIP)
