# Supplementary figures and images for: Comprehensive Analysis of Mandibular Residual Asymmetry after Bilateral Sagittal Split Ramus Osteotomy Correction of Menton Point Deviation
Source: PLoS One. 2016 Aug 29;11(8):e0161601. doi: 10.1371/journal.pone.0161601 (PMC5003338; doi:10.1371/journal.pone.0161601)

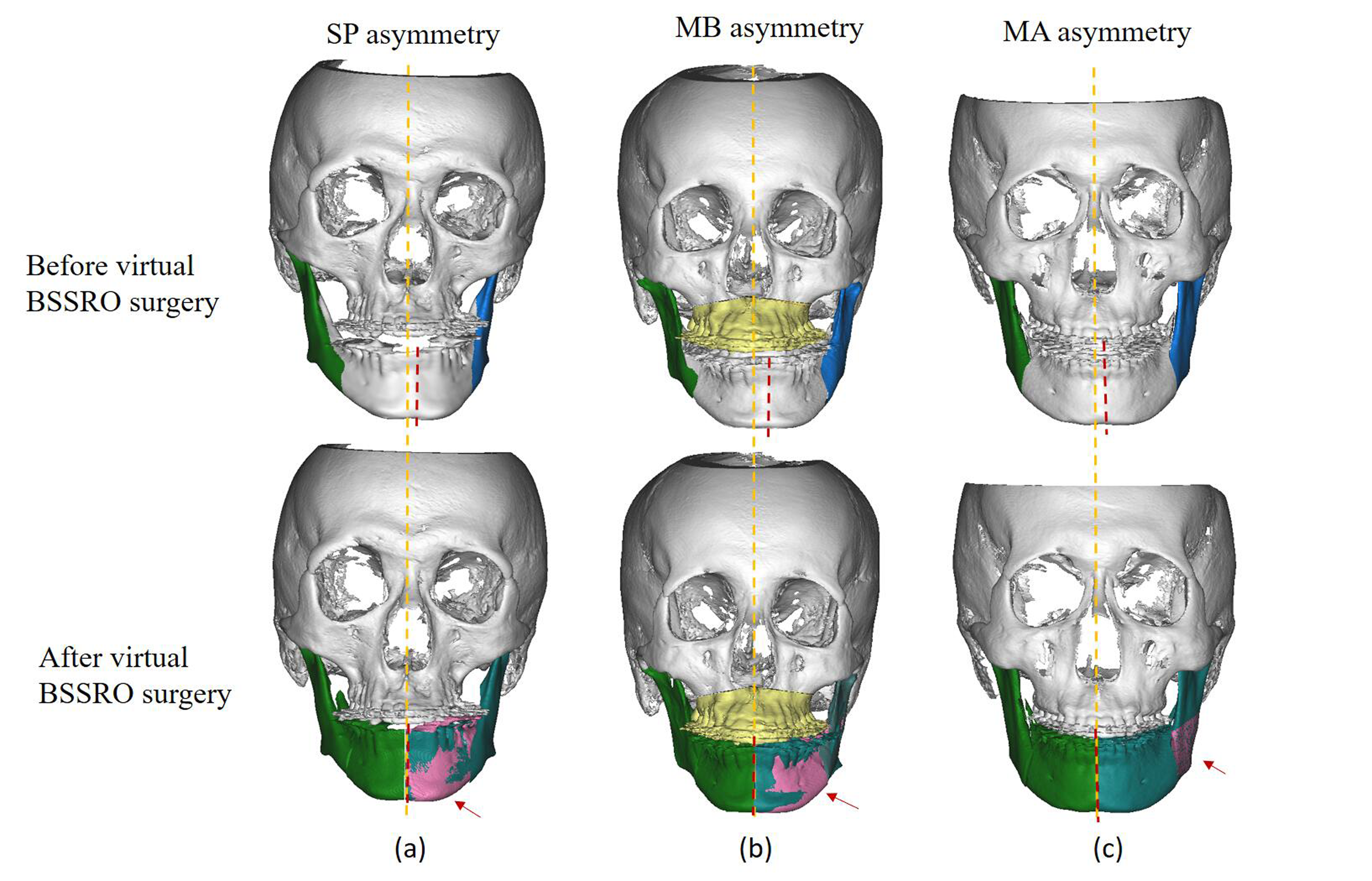

Supplement: S1 Fig — the three models in the first row represent the stage before virtual BSSRO surgery (BSSRO with/without Lefort I operation). The second row of the models represent after virtual Me point correction, and mirroring and superimposition of the hemi-mandible. Pink color represents the residual asymmetry between both sides of the mandible. (a) SP region asymmetry: asymmetry mainly exist in the SP region; (b) MB region asymmetry: asymmetry mainly exist in the MB region; (c) MA region asymmetry: asymmetry mainly exist in the MA region. (TIF) [file pone.0161601.s001.tif]

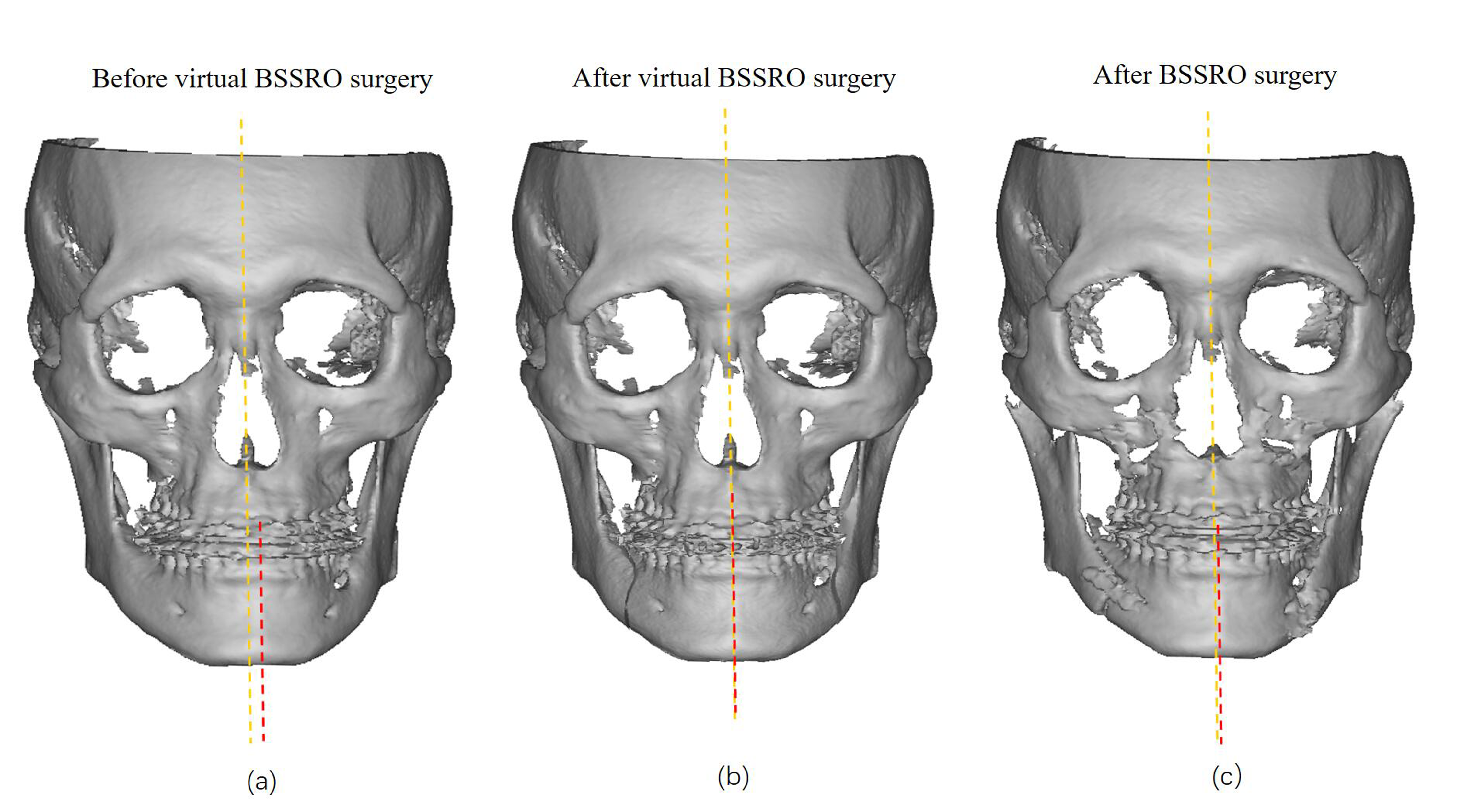

Supplement: S2 Fig — (a) Three dimensional model with pre-surgical orthodontic treatment (before virtual BSSRO surgery); (b) Three dimensional model after virtual BSSRO surgery; (c) Three dimensional post-surgical model. The volumetric differences between both sides of hemi-mandible were calculated to verify the outcome consistency between surgery and virtual surgery. (TIF) [file pone.0161601.s002.tif]
