# Supplementary material for: Comprehensive Analysis of Mandibular Residual Asymmetry after Bilateral Sagittal Split Ramus Osteotomy Correction of Menton Point Deviation
Source: PLoS One. 2016 Aug 29;11(8):e0161601. doi: 10.1371/journal.pone.0161601 (PMC5003338; doi:10.1371/journal.pone.0161601)
Supplement: S1 Table — (DOCX) [file pone.0161601.s003.docx]

**S1 Table.** **The mean values of clinical examination after virtual Me point correction and after clinical orthognathic surgery.**

| Variable | Virtual Me point correction group | Post-surgical group |
| --- | --- | --- |
| Deviation of Me point (mm) | 5.4±0.9 | 1.0±0.4 |
| Deviation of dental midline (mm) | 2.9±0.6 | 0.7±0.3 |
| Incidence of inclined maxillary occlusal plane (%) | 29.1 (n=16) | 0 |
| Inclination of occlusal plane (°) | 6.1±1.3 | 1.0±0.4 |
| ANB angle (°) | -5.1±1.7 | 2.7±1.0 |
